# Evaluation of Korean-Language COVID-19–Related Medical Information on YouTube: Cross-Sectional Infodemiology Study

**DOI:** 10.2196/20775

**Published:** 2020-08-12

**Authors:** Hana Moon, Geon Ho Lee

**Affiliations:** 1 Department of Family Medicine School of Medicine Daegu Catholic University Daegu Republic of Korea

**Keywords:** COVID-19, YouTube, social media, misinformation, public health surveillance, health communication, consumer health information, health education, infectious disease outbreaks, infodemiology, infoveillance, infodemic, internet, multimedia

## Abstract

**Background:**

In South Korea, the number of coronavirus disease (COVID-19) cases has declined rapidly and much sooner than in other countries. South Korea is one of the most digitalized countries in the world, and YouTube may have served as a rapid delivery mechanism for increasing public awareness of COVID-19. Thus, the platform may have helped the South Korean public fight the spread of the disease.

**Objective:**

The aim of this study is to compare the reliability, overall quality, title–content consistency, and content coverage of Korean-language YouTube videos on COVID-19, which have been uploaded by different sources.

**Methods:**

A total of 200 of the most viewed YouTube videos from January 1, 2020, to April 30, 2020, were screened, searching in Korean for the terms “Coronavirus,” “COVID,” “Corona,” “Wuhan virus,” and “Wuhan pneumonia.” Non-Korean videos and videos that were duplicated, irrelevant, or livestreamed were excluded. Source and video metrics were collected. The videos were scored based on the following criteria: modified DISCERN index, Journal of the American Medical Association Score (JAMAS) benchmark criteria, global quality score (GQS), title–content consistency index (TCCI), and medical information and content index (MICI).

**Results:**

Of the 105 total videos, 37.14% (39/105) contained misleading information; independent user–generated videos showed the highest proportion of misleading information at 68.09% (32/47), while all of the government-generated videos were useful. Government agency–generated videos achieved the highest median score of DISCERN (5.0, IQR 5.0-5.0), JAMAS (4.0, IQR 4.0-4.0), GQS (4.0, IQR 3.0-4.5), and TCCI (5.0, IQR 5.0-5.0), while independent user–generated videos achieved the lowest median score of DISCERN (2.0, IQR 1.0-3.0), JAMAS (2.0, IQR 1.5-2.0), GQS (2.0, IQR 1.5-2.0), and TCCI (3.0, IQR 3.0-4.0). However, the total MICI was not significantly different among sources. “Transmission and precautionary measures” were the most commonly covered content by government agencies, news agencies, and independent users. In contrast, the most mentioned content by news agencies was “prevalence,” followed by “transmission and precautionary measures.”

**Conclusions:**

Misleading videos had more likes, fewer comments, and longer running times than useful videos. Korean-language YouTube videos on COVID-19 uploaded by different sources varied significantly in terms of reliability, overall quality, and title–content consistency, but the content coverage was not significantly different. Government-generated videos had higher reliability, overall quality, and title–content consistency than independent user–generated videos.

## Introduction

The coronavirus disease (COVID-19) is rapidly spreading all over the world. South Korea has noticeably flattened the curve of infection and recorded low fatality rates despite an explosion of cases after the 31st patient was confirmed [1]. The South Korean government’s key strategies were transparency in sharing information, mass screening, tracking of suspected cases, and the reallocation of medical resources [1].

However, without public cooperation, these governmental measures would not have been effective. South Korea’s success, thus far, prompts us to seek answers to the following questions:

- “What explains the early adoption of social distancing among Koreans?”
- “Why did they come to accept the cost to their privacy and voluntarily cooperate with authorities?”
- “What persuaded them to change their behaviors in such a short time?”

YouTube may have served as a valuable tool in providing information on COVID-19, creating understanding among South Koreans and urging them to cooperate with the authorities in implementing precautionary measures. South Korea is one of the best connected countries in the world, with 88.5% of the population accessing the internet via smartphones in 2019 [2]. Even on public transportation such as buses, subways, and trains, South Koreans enjoy free public Wi-Fi. Thus, commuters can easily access online video platforms without worrying about their phone bill. YouTube has become the most popular video platform in South Korea in terms of monthly average use time [3]. Bearing in mind that health conscious consumers often search for health information online, South Koreans may have accessed YouTube for information on COVID-19 [4]. Therefore, due to its popularity, YouTube may have provided timely and relevant information on COVID-19 to the public.

The popularity of a YouTube video has been the focus of marketing researchers since the platform became one of the leading types of advertising media [5]. Researchers primarily ask, “What key factors determine video popularity?” Many factors have been suggested, such as the video’s title, thumbnail, subtitles, video upload date and time, delivery style, running time, or social network of the YouTube channel [6]. There have also been attempts to build computer-based video popularity prediction models [7-9]. However, the measurement of popularity is not easy to determine because video metrics are constantly evolving. For example, it seems reasonable to consider a video clip with 1 million views in 1 week popular compared to a video with the same views that have been accumulating for 5 years. In medical informatics, the video power index (VPI) has been proposed to solve this problem using a simple formula [10]. VPI captures the views, likes, and dislikes over the number of days that the video has been posted.

In contrast with marketing researchers who have studied social media to promote their brand or merchandise, medical researchers have been using social media for public health surveillance [11]. Social media can be used as a tool to provide a snapshot of the public’s interest in and response to ongoing health issues [12]. A wide variety of health topics have been analyzed so far, including infectious diseases, mental disorders, and chronic diseases [11]. For example, previous studies have quantitatively analyzed Twitter data to assess people’s attitudes toward mental illness [13,14]. In addition to YouTube, various sources were used to retrieve health-related data, including search engines, blogs, forums, and social media platforms such as Google, Bing, Baidu, Yahoo, Twitter, and Facebook [15]. The frequency of relevant keywords or trending hashtags, as well as numbers of views, likes, and shares, is typically measured. Manual coding and computer-based methods including content analysis, text mining, natural language processing, and topic modeling have been used to determine the most talked about topics [11,16]. Moreover, there were also attempts to predict infectious disease outbreaks or quickly detect a person who has depression. Demand-based infoveillance studies using internet search queries have primarily focused on predicting infectious disease outbreaks, such as Zika, influenza, dengue, and the measles virus [17-19]. Other studies analyzed user’s behavior on social media and proposed a model that was based on machine learning for the early detection of depression and suicidal risk [20,21].

Content analysis has been widely used in YouTube studies involving online medical information. The question of who is supplying what information has been addressed in various fields of research, including ulcerative colitis, tinnitus, sleep apnea, cervical cancer, and orthodontics [22-26]. In contrast with Twitter studies that use computer-assisted content coding, the content in these studies was extracted from videos and manually coded. Notably, the medical information and content index (MICI) has been a commonly used tool in infectious disease studies since it was proposed to systematically assess the content coverage of Ebola hemorrhagic fever [27]. MICI assesses five key components for understanding infectious disease: (1) prevalence, (2) transmission, (3) clinical symptoms, (4) screening and testing, and (5) treatment and outcome.

Recently, YouTube has garnered attention from researchers in medical communication and education. Traditionally, written medical information has been criticized for its low accessibility. Extensive use of medical jargon, with which laypeople may be unfamiliar, hinders the delivering of complete messages [28]. Such accessibility problems have also been a barrier in informed or shared decision making [29]. In contrast, YouTube videos offer easy-to-understand information because videos can contain multimedia elements such as graphics, animations, and voice-overs using verbal expressions. In this regard, YouTube could be a user-friendly tool to educate the public on health-related topics.

However, there are concerns about the reliability and quality of online information. Viewers may be exposed to misinformation because YouTube does not have a verification process that videos must pass before being published. The spread of misinformation via social media is amplified by the filter bubble and the echo chamber effect. Many people access news from their social media feeds, which are curated by an algorithm for each person. This filtered information exposes users less to opposing viewpoints and isolates them in their own bubbles [30]. Furthermore, the echo chamber effect means that people prefer to read articles that confirm and strengthen their original opinions. Users also prefer to interact with like-minded people, allowing misinformation more opportunities to go viral [31]. It is the responsibility of each YouTube user to be aware of the veracity of the information they watch and share.

Moreover, the reliability and quality of medical information are of the highest importance. Inaccurate information may lead to physical harm or irreversible damage. Medical misinformation may have life-threatening consequences for vulnerable populations such as patients with cancer, children, and older people. For example, patients with cancer taking alternative medicine are more likely to refuse evidence-based therapies and have higher mortality rates than patients who do not take alternative medicine [32]. Moreover, thriving antivaccine movements on social media have made parents hesitant to have their children vaccinated, possibly contributing to a reduction in vaccination rates and leading to multiple measles outbreaks [33].

Researchers, therefore, have tried to compare misleading and useful information as well as evaluate the reliability and quality of consumer medical information. Some studies have attempted to examine the differences in video metrics between misleading and useful videos, including the number of views, likes, dislikes, and running time [34-37]. The results have been controversial. Various measurement tools have been suggested to evaluate the reliability and quality of consumer medical information, such as DISCERN, Health on the Net code, the Journal of the American Medical Association Score (JAMAS) benchmark criteria, and the global quality score (GQS) [10,38-43]. Other studies have compared videos based on their sources (ie, government agencies, news agencies, health care professionals, and independent users) and then on the reliability and quality of these sources. Their results indicate concerns about the reliability of information that is neither monitored nor filtered. Although government- or professionally generated videos were more likely to be reliable and accurate, they were falling behind in nurturing their YouTube presence [34,44,45]. In contrast, individual user-generated videos were superior in number [35]; however, they were more likely to be inaccurate and incomplete [46]. Consequently, consumers are exposed to misleading medical information.

The impact of disseminating misinformation is particularly important in the context of public health emergencies. False beliefs or misperceptions disseminated via YouTube can spread mistrust toward authorities, generate confusion, and heighten public anxiety. Furthermore, fake news can cause people to engage in undesirable behaviors such as panic buying of food, medicines, and toilet paper due to fears about the pandemic [47]. Panic buying and hoarding can make it difficult for physically challenged or older people to buy essential products. Therefore, several studies have analyzed the spread of medical information through YouTube videos on outbreaks of several infectious diseases such as the H1N1 influenza, Ebola virus disease, and Zika virus disease [27,35,48,49].

Social media has been analyzed as a source of information on infectious disease in a number of studies; however, there is limited research on COVID-19. Twitter and Weibo have been analyzed to understand the impacts of COVID-19 and social distancing on mental health [50,51]. Although several studies have analyzed the content of COVID-19 videos in English, Spanish, and Chinese, this study is, as far as we are aware, the first to evaluate the Korean-language content of COVID-19 videos on YouTube [36,44,52]. Korean, which is a distinct language and not a dialect of Chinese or Japanese, is the only official language of Korea; therefore, it is important to evaluate the Korean video content.

This study investigated three main research questions:

1. Are there differences in video metrics between misleading and useful Korean-language videos about COVID-19 on YouTube?
2. Do Korean-language YouTube videos on COVID-19 that are uploaded by different sources vary significantly in reliability, overall quality, title–content consistency, and content coverage?
3. Do government videos have higher reliability, overall quality, title–content consistency, and content coverage compared to independent user–generated videos?

## Methods

### Recruitment

Data were obtained from publicly available YouTube videos. A total 200 of the most extensively viewed videos were screened from the YouTube video application programming interface. The search criteria used to obtain the videos comprised of the following terms in Korean: “Coronavirus,” “COVID,” “Corona,” “Wuhan virus,” and “Wuhan pneumonia.” Although the authors are aware that the last two keywords are inappropriate as they could create the impression of discrimination, these terms had to be included in the search to identify the videos uploaded on YouTube during the initial phase of the COVID-19 pandemic from January to early February 2020. Prior to the World Health Organization announcing the official name of the virus on February 11, 2020, “Wuhan virus” and “Wuhan pneumonia” were the commonly used terms describing the virus on social media [53].

Videos uploaded from January 1, 2020, to April 30, 2020, were included. Exclusion criteria were videos in languages other than Korean and videos that were duplicated, irrelevant, or livestreamed. Date of upload; source; number of views, likes, dislikes, and comments; the view ratio (number of views / days); and the likes ratio (likes ∗ 100 / [likes + dislikes]) as of May 1, 2020, were collected.

**Figure 1:**
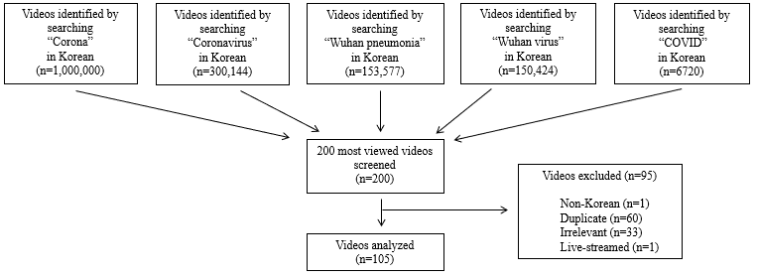
Data collection flow.

As shown in Figure 1, a total of 1,610,865 videos were obtained. Of the 200 most widely viewed videos, 95 videos were excluded based on the exclusion criteria. A total of 105 videos with 126,633,036 views were included in the study. Of the 105 included videos, 47 (44.76%) were from independent users, 39 (37.14%) were from news agencies, and 11 (10.48%) were from health care professionals. Government agencies contributed only 8 (7.62%) of the total videos.

### Ethics Statement

This study was reviewed and approved as exempt research by the corresponding author’s Institutional Review Board (CR-20-102-L). 

### Assessment of Popularity

The VPI was used to assess video popularity [10]. The formula for VPI is the (ratio of likes * ratio of views / 100). The view ratio (number of views / days); and the likes ratio (likes ∗ 100 / [likes + dislikes]) as of May 1, 2020, were assessed.

### Categorization of Source

Videos were categorized based on their source, which comprised of government agencies, news agencies, health care professionals, and independent users. Government agencies include the Korea Centers for Disease Control and Prevention (KCDC), central disaster management headquarters, Cheongwadae (the executive office and official residence of the South Korean president), the regional health departments, medical associations, academic institutions, and hospitals. News agencies include news clips from newspapers or a broadcast television network. Health care professionals include physicians, nurses, pharmacists, or other health care providers, who do not represent the opinion of their affiliation. Independent users are individuals who are not health care professionals and have no established affiliation, and we included YouTubers from both Korea and other countries who made Korean-language videos.

### Assessment of Usefulness

Videos were classified as useful or misleading, and useful videos were defined as those with scientifically correct information. This study’s authors are physicians who have provided patient care in Korea during the COVID-19 pandemic and are aware of the uncertainty regarding the disease; therefore, KCDC guidelines as of May 1, 2020, were used as a standard to determine whether a video’s content was scientifically correct. Misleading videos were defined as those with scientifically unproven information, and we considered a video misleading if it contained any misleading information at all because it still had the potential to disseminate misinformation. The complete list of misleading information is available in Textbox 1.

**Detailed statements about misleading videos.**

• The coronavirus disease (COVID-19) outbreak is not a concern for the public.

• COVID-19 does not exist, and it is a hoax perpetrated to make money.

• COVID-19 has been weaponized.

• Conspiracy theories are concerned with where it originated, how it spread, and who was responsible for it.

• Authorities are hiding the truth.

• COVID-19 was manufactured with the goal of human depopulation.

• One type of food or supplement is recommended over another as a cure or for the prevention of COVID-19.

• COVID-19 has occurred as part of nature’s revenge on humankind for cruelty to wild animals.

• People are recommended to not follow the guidelines of the Korea Centers for Disease Control and Prevention.

• People are recommended to follow scientifically unproven measures.

• The COVID-19 pandemic was prophesied by clairvoyants.

• COVID-19 was started to insert microchips into people under the guise of a vaccine.

• COVID-19 was created to build a global surveillance system.

### Assessment of Reliability

Videos classified as useful were further analyzed for reliability, overall quality, title–content consistency, and content. There is still no consensus on how to evaluate medical information contained in videos; therefore, we adopted the evaluation tools commonly used in previous studies on online consumer health information, such as tools for websites (JAMAS, GQS) and written patient information on treatment choices (DISCERN) [10,27,36,39,41-43]. The reliability of videos was determined using both the modified DISCERN index and the JAMAS benchmark criteria. DISCERN assesses clarity, reliability, bias, reference supplementation, and areas of uncertainty [36,42]. One point is awarded for each criterion, and the maximum total score is 5 points. JAMAS benchmark criteria consist of authorship, attribution, disclosure, and current status [10,42,43]. One point is awarded for each criterion, and the maximum total score is 4 points. The full list of the modified DISCERN index and JAMAS benchmark criteria is available in Multimedia Appendices 1 and 2, respectively.

### Assessment of Overall Quality

The overall quality of the videos was determined by the GQS, which is a 5-point Likert scale ranging from 1 (poor quality) to 5 (high quality) [10,41,43]. It consists of the flow of information, ease of use, and usefulness. The full list of GQS criteria is available in Multimedia Appendix 3.

### Assessment of Title–Content Consistency

We created a novel scoring system for assessing title–content consistency because there was no validated evaluation tool for this. The title–content consistency index (TCCI) is a 5-point Likert scale ranging from 1 (poor consistency) to 5 (high consistency), which rates the sensationalist style of a video, that is, the gap between title and content. This index was developed based on previous research on junk news and clickbait [53,54]. The full list of TCCI is available in Multimedia Appendix 4.

### Content Analysis

We used the MICI for content analysis [27,36]. MICI is a 5-point Likert scale to assess five components of medical information: (1) prevalence, (2) transmission and precautionary measures, (3) signs and symptoms, (4) testing, and (5) treatment and outcome. The full list of MICI is available in Multimedia Appendix 5.

### Statistical Analysis

All videos were reviewed and evaluated by two independent authors. Discrepancies between the authors were resolved by discussion. Interreviewer agreement for categorical variables was analyzed using the Cohen kappa coefficient. Interreviewer agreement for continuous variables was analyzed using intraclass correlation coefficient (ICC) estimates and their 95% confidence intervals based on average measures, absolute agreement, and two-way random model. The kappa coefficients of agreement regarding the classification of source and the usefulness of the videos were k=1 in both cases, indicating full agreement between the authors. ICCs regarding DISCERN, JAMAS, GQS, TCCI, and MICI were 0.47 (95% CI –0.29 to 0.81), 0.93 (95% CI 0.80 to 0.98), 0.71 (95% CI 0.17 to 0.90), 0.89 (95% CI 0.70 to 0.96), and 0.91 (95% CI 0.73 to 0.97), respectively. The Shapiro-Wilk test was used to assess the normality of data. Normally distributed continuous variables were presented as mean (SD). Nonnormally distributed continuous variables were presented as median (IQR). Categorical variables were compared by chi-square test or Fisher exact tests and presented as a number (percentage). Student *t* test for continuous variables and chi-square test for categorical variables were used to compare misleading and useful videos. Kruskal-Wallis test and Mann-Whitney test with Bonferroni correction were used to compare the four sources. All analyses were conducted with the R statistical package version 3.6.3 (R Foundation for Statistical Computing). A *P* value<.05 was considered statistically significant.

## Results

### Misleading Versus Useful Video

The characteristics of misleading and useful videos are contained in Table 1. Of the total videos, 37.14% (39/105) contained misleading information. Independent user–generated videos showed the highest proportion of misleading information at 68.09% (32/47), while all the government-generated videos were useful. The mean number of likes was 1.47 times higher in misleading videos (18,266 vs 12,389, *P*=.03). The mean number of comments was 1.42 times higher in useful videos (2203 vs 3224, *P*=.02). Misleading videos had almost twice the running time than useful videos (795 seconds vs 405 seconds, *P*=.03). There was no significant difference in the mean views, dislikes, and VPI between the two groups (*P*=.11, *P*=.08, *P*=.31, respectively).

**Table 1:** The characteristics of misleading and useful videos.^a^

Variables^b^		Misleading videos	Useful videos	Total	*P* value
Videos, n (%)		39 (37.14)	66 (62.86)	105 (100)	N/A^c^
**Video metrics, mean (SD)**					
	Views	972,020 (800,267)	1,344,307 (1,537,507)	1,206,029 (1,320,654)	.11
	Likes	18,266 (12,772)	12,389 (13,519)	14572 (13,490)	.03
	Dislikes	774 (656)	545 (622)	630 (641)	.08
	Comments	2203 (1532)	3224 (2983)	2845 (2581)	.02
	Days	79 (22)	70 (27)	73 (26)	.09
	Length (seconds)	795 (1046)	405 (340)	550 (713)	.03
	VPI^d^	67,708 (159,243)	129,469 (443,911)	106,529 (365,137)	.31
**Source, n (%)**					
	Independent users	32 (68.09)	15 (31.91)	47 (44.76)	N/A
	News agencies	4 (10.26)	35 (89.74)	39 (37.14)	N/A
	Health care professionals	3 (27.27)	8 (72.73)	11 (10.48)	N/A
	Government agencies	0 (0.00)	8 (100)	8 (7.62)	N/A

^a^Student *t* test was used to compare misleading and useful videos.

^b^Continuous variables were presented as mean (SD), and categorical variables were presented as n (%).

^c^N/A: not applicable.

^d^VPI: video power index.

### Video Metrics, Reliability, Overall Quality, and Title–Content Consistency of the Useful Videos by Source

Video metrics, reliability, overall quality, title–content consistency, and content of the useful videos by source are presented in Table 2. The distribution of views, likes, comments, and length was significantly different across the four sources (independent users: *P*=.04, news agencies: *P*=.005, health care professionals: *P*=.03, and government agencies: *P*=.002). Videos by government agencies had the shortest median running time of 41 seconds, with the highest views. VPI, as a measurement of popularity, was not significantly different among sources. Government agency–generated videos achieved the highest median score of DISCERN (5.0, IQR 5.0-5.0), JAMAS (4.0, IQR 4.0-4.0), and GQS (4.0, IQR 3.0-4.5), while independent user–generated videos achieved the lowest median score of DISCERN (2.0, IQR 1.0-3.0), JAMAS (2.0, IQR 1.5-2.0), and GQS (2.0, IQR 1.5-2.0). These differences were statistically significant (*P*<.001, *P*<.001, *P*<.001, respectively). The median scores of TCCI were 3.0 (IQR 3.0-4.0) in independent users, 5.0 (IQR 4.0-5.0) in news agencies, 3.0 (IQR 2.0-4.0) in health care professionals, and 5.0 (IQR 5.0-5.0) in governmental agencies (*P*<.001).

**Table 2:** Video metrics, reliability, overall quality, title–content consistency, and content of the useful videos by source.

Variables^a^			Independent users	News agencies	Health care professionals	Government agencies	Total	*P* value^b^
Videos, n (%)			15 (22.73)	35 (55.03)	8 (12.12)	8 (12.12)	66 (100.00)	N/A^c^
**Video metrics, median (IQR)**								
	Views		744,824 (553,149-1,050,672)	906,731 (768,863-1,282,426)	928,563 (768,613-1,083,527)	2,418,742 (856,649-3,896,445)	888,772 (726,536-1,292,581)	.04
	Likes		9643 (5063-18,041)	7523 (4727-12,831)	20,874 (14,977-27,702)	1037 (157-15,169)	9756 (4598-16,432)	.005
	Dislikes		348 (190-494)	305 (195-469)	789 (396-894)	104 (19-1280)	355 (179-698)	.10
	Comments		1251 (779-2516)	2938 (1810-5570)	2031 (1055-3242)	179 (35-3921)	2377 (1251-4151)	.03
	Days		56 (32-84)	85 (47-98)	72 (67-94)	74 (54-82)	75 (49-96)	.22
	Length (seconds)		422 (246-518)	207 (165-536)	791 (371-822)	41 (36-176)	249 (162-602)	.002
	VPI^d^		15,689 (5786-112,775)	22,326 (4568-50,043)	33,709 (16,773-54,613)	9652 (755-208,260)	18,092 (5780-56,760)	.54
**Reliability, median (IQR)**								
	DISCERN		2.0 (1.0-3.0)	4.0 (4.0-5.0)	4.5 (3.0-5.0)	5.0 (5.0-5.0)	4.0 (3.0-5.0)	<.001
	JAMAS^e^		2.0 (1.5-2.0)	3.0 (3.0-4.0)	2.5 (2.0-3.0)	4.0 (4.0-4.0)	3.0 (2.0-4.0)	<.001
**Overall quality, median (IQR)**								
	GQS^f^		2.0 (1.5-2.0)	3.0 (2.0-3.0)	3.5 (2.5-4.5)	4.0 (3.0-4.5)	3.0 (2.0-3.0)	<.001
**Title-content consistency, median (IQR)**								
	TCCI^g^		3.0 (3.0-4.0)	5.0 (4.0-5.0)	3.0 (2.0-4.0)	5.0 (5.0-5.0)	5.0 (3.0-5.0)	<.001
**Content**								
	**Frequency, n (%)**							
		Prevalence	10 (66.67)	27 (77.14)	5 (62.50)	2 (25)	44 (66.67)	N/A
		Transmission and precautionary measures	11 (73.33)	23 (65.71)	7 (87.50)	8 (100)	49 (74.24)	N/A
		Signs and symptoms	4 (26.67)	17 (48.57)	3 (37.50)	7 (87.50)	31 (46.97)	N/A
		Testing	7 (46.67)	15 (42.86)	4 (50)	4 (50)	30 (45.45)	N/A
		Treatment and outcome	3 (20)	16 (45.71)	4 (50)	1 (12.50)	24 (36.36)	N/A
	Total score of MICI^h^, median (IQR)		5.0 (3.0-7.0)	5.0 (3.5-6.5)	7.0 (2.5-8.0)	5.0 (5.0-9.0)	5.0 (3.00-7.0)	.77
	**Individual scores of the MICI components, median (IQR)**							
		Prevalence	1.0 (0.0-1.0)	1.0 (1.0-1.0)	1.0 (0.0-2.5)	0.0 (0.0-0.5)	1.0 (0.0-1.0)	.18
		Transmission and precautionary measures	2.0 (0.5-3.0)	2.0 (0.0-3.0)	2.5 (1.5-3.5)	3.0 (2.0-3.5)	2.0 (0.0-3.0)	.23
		Signs and symptoms	0.0 (0.0-1.0)	0.0 (0.0-2.0)	0.0 (0.0-2.0)	2.0 (2.0-2.5)	0.0 (0.0-2.0)	.03
		Testing	0.0 (0.0-2.0)	0.0 (0.0-2.0)	0.5 (0.0-1.0)	1.0 (0.0-2.5)	0.0 (0.0-2.0)	.86
		Treatment and outcome	0.0 (0.0-0.0)	0.0 (0.0-2.0)	0.5 (0.0-2.0)	0.0 (0.0-0.0)	0.0 (0.0-1.0)	.23

^a^Continuous variables were presented as median (IQR), and categorical variables were presented as n (%).

^b^Kruskal-Wallis tests were used to calculate *P* values.

^c^N/A: not applicable.

^d^VPI: video power index.

^e^JAMAS: Journal of the American Medical Association Score.

^f^GQS: global quality score.

^g^TCCI: title–content consistency index.

^h^MICI: medical information and content index.

### Content Analysis of the Useful Videos by Source

Of the useful videos, 74.24% (49/105) provided information on “transmission and precautionary measures,” 66.67% (44/105) contained information on “prevalence,” 46.97% (31/105) contained “signs and symptoms,” 45.45% (30/105) contained “testing,” and 36.36% (24/105) contained “treatment and outcome.” “Transmission and precautionary measures” were the most discussed topic by government agencies, news agencies, and independent users. Every video by government agencies covered “transmission and precautionary measures,” and 7 out of 8 (87.50%) videos mentioned “signs and symptoms.” On the other hand, the most mentioned topic by news agencies was “prevalence,” followed by “transmission and precautionary measures.” The total score of MICI was not significantly different among sources (*P*=.77). The highest median score among individual MICI components was shown in “transmission and precautionary measures” of government agency–generated videos (3.0, IQR 2.0-3.5).

## Discussion

### Principal Findings

This study is the first of its kind to evaluate the Korean-language content of COVID-19 videos on YouTube. Previous COVID-19 YouTube studies have captured videos using a relevant filter at the time of the search or have been mainly descriptive [36,52]. In this study, we analyzed the 200 most popular videos uploaded between January 1, 2020, and April 30, 2020, which comprised 126,633,036 views. We conducted content analysis and assessed the reliability, overall quality, and title–content consistency of the videos.

One must be cautious when labeling content as misinformation; however, the majority of YouTube studies in the field of emerging infectious disease did not show detailed criteria or specific examples of misleading videos. Some of them showed two or three examples of conspiracy theories, and others merely mentioned that they classified videos as misleading if they conveyed at least one scientifically unproven piece of information [35,55,56]. In contrast, one study on the Ebola virus provided specific examples of misleading videos [46]. COVID-19–related YouTube studies are not unlike previous studies on other infectious diseases, and one briefly mentioned that they reviewed published references as the standard for what is known about COVID-19 [57]. Notably, a study performed by Li et al [44] provided statements recorded from YouTube videos classified as misleading. Li et al [44] also created a novel five-point scoring system to assess the usefulness of a video. However, this score is not designed to distinguish misleading videos from useful ones but to measure how much of the video content is useful. Unlike previous studies, we set clear criteria to distinguish misleading from useful videos and provided a complete list of misleading information in Textbox 1.

Out of 105 videos, 39 (37.14%) were found to be misleading. This percentage is higher than that from a previous study that evaluated English-language videos addressing COVID-19 (19/69, 27.5%) [44]. Useful videos did not exceed misleading ones in popularity, which suggests that the chance of a layperson being exposed to inaccurate information is quite high. Unfortunately, fake news spreads six times faster than verified news and receives higher viewer interaction [58,59]. Biggs et al [37] reported that misleading videos are more viewed than useful videos because the useful ones have longer running times; however, recent studies on COVID-19–related YouTube videos have returned inconsistent results. Although useful videos gained more views than misleading ones in this study, the difference was not statistically significant (1,344,307 vs 972,020, *P*=.11). Furthermore, useful videos earned more comments but fewer likes than misleading videos (3224 vs 2203, *P*=.02 and 12,389 vs 18,266, *P*=.03, respectively). Previously, there was an attempt to compare Mandarin videos regarding COVID-19 to English ones. They reported that misleading Mandarin videos gained more views than useful ones, but the result was the opposite for English videos (Mandarin: 91,949 in useful videos, 151,868 in misleading videos, *P*=.30; English: 288,545 in useful videos, 1621 in misleading videos, *P*<.001) [36]. Another study on English-language COVID-19–related videos found that there were no significant differences in views, likes, and dislikes between useful and misleading videos (*P*=.50, *P*=.79, *P*=.10, respectively) [44].

In this study, most of the misleading information was delivered by independent users (32/39, 82.05%). Moreover, some of these videos generated a lot of interaction from viewers. For example, one video suggested that because of COVID-19, a microchip will be inserted into people under the guise of a vaccine to build a global surveillance system; this video gained 330,672 views with 2454 comments. Another video mentioned a conspiracy theory that COVID-19 is a biological weapon, and it was manufactured for human depopulation. Another video with 1,478,262 views claimed that COVID-19 was predicted in works of fiction or in movies. One video posted by a shaman was entirely misleading. It consisted of question-answer pairs, and a shaman answered questions about COVID-19 such as, “When will the COVID-19 pandemic end?” and “When will a COVID-19 vaccine be available?”

The mere fact that a video uploader is a doctor or health expert does not imply that their videos provide accurate medical information. There were 3 out of 11 videos posted by health care professionals that were misleading; 2 videos addressed misleading information throughout the entire running time. There was 1 video that alleged the existence of a conspiracy theory as to when COVID-19 originated and who is responsible for the virus. Another video recommended one vitamin supplement as a cure for COVID-19. In contrast, one video posted by a pharmacist provided partially useful information, but it was still classified as misleading since videos containing any misleading information could potentially disseminate misinformation. This video included helpful information during the first half of the running time, such as characteristics of the viral disease, transmission, hand hygiene, and face masks. However, during the second half, the video recommended several foods such as ginger, onions, green tea, and black beans as immune boosters against COVID-19. Although good nutrition is key to staying healthy, this video was classified as misleading because no food or dietary supplement alone can prevent COVID-19.

Most videos by news agencies in this study provided scientifically accurate information (35/39, 89.74%). Video clips of television-based news were often posted on YouTube, amplifying the impact of traditional media. Given the inclusion criterion for this study, it can be reasoned that consumers may have the social media literacy skills to choose appropriate videos among the hundreds of thousands of videos that can be viewed on YouTube.

Government-generated videos were effective delivery tools. Although they comprised only 7.62% (8/105) of the total videos, they were all useful and gained the highest median views (*P*=.04). They also had the shortest median running time at 41 seconds (*P*=.002) and showed higher reliability and overall quality (all *P*<.001). These findings are consistent with previous studies. A systematic review of health care information on YouTube found that government agency–generated videos had credible information [45]. Similarly, in a study performed by Li et al [44], government videos only contributed 2.89% (2/69) of COVID-19–related English videos, but they contained only useful information and showed higher reliability compared to consumer videos (DISCERN: 4.57 vs 2.12, *P*=.008; JAMAS: 2.71 vs 1.50, *P*=.03) [44].

However, credible videos with high quality were not popular. Considering that videos generated by government agencies received the least number of likes and comments (*P*=.005, *P*=.03, respectively), they failed to encourage viewer interaction and engagement. They also showed the lowest VPI as a measurement of popularity, but the VPI was not significantly different (*P*=.54). Government or news agencies were also more likely to post videos with a proper title (median TCCI of 5.0, IQR 4.0-5.0 and 5.0, IQR 5.0–5.0, respectively). In contrast, independent users were more likely to post clickbait videos with sensationalist headlines or eye-catching, attention-grabbing thumbnails with large gaps between the title and their content (median TCCI 3.0, IQR 3.0-4.0). Viewers are more likely to select emotionally appealing titles, regardless of the correctness of the content [60].

Several studies have reported content differences among analyzed COVID-19–related YouTube videos. Basch et al [52] reported that “quarantine and travel restrictions” was the most discussed item in English and Spanish videos (89/89 and 84/89, respectively), and “precautionary measures” was covered in less than one-third (0/100 to 31/100) of the videos. In a study performed by Khatri et al [36], only 10% (2/21) of Mandarin videos covered “testing” compared to 53.19% (25/47) of the English videos [36]. In our study, 45.45% (30/66) of videos covered “testing.” On the other hand, “transmission” was the most mentioned subject in both Mandarin and English videos, which is similar to our finding (49/66, 74.24% in our study; 43/47, 91.49% in English; 17/21, 81% in Mandarin).

Through content analysis, we can understand the characteristics of popular COVID-19–related videos in Korea. As shown in Table 2, the most common content was “transmission and precautionary measures.” Even all the videos published by the government agencies covered “transmission and precautionary measures.” Various personal protective strategies were emphasized in these videos, including washing hands, wearing a mask, maintaining a distance of 1-2 meters, staying home when sick, and avoiding gatherings. These strategies may encourage people to practice preventive behaviors such as personal hygiene and social distancing. Asymptomatic carriers were also mentioned in these videos, and the comments on these videos show that people share similar concerns. For example:

I am young and healthy. It looks like I will probably be okay, but what if I spread the virus to my parents without knowing it? They are old and weak. I’ve got to stay at home.

The second most common content area in Korean videos regarding COVID-19 covered “signs and symptoms.” These videos may encourage people who develop suspicious symptoms to be screened as soon as possible. Early detection of the symptoms of COVID-19 may enable people to visit the hospital early on, improving treatment responses and leading to decreased mortality.

Furthermore, videos frequently mentioned call centers run by KCDC or the regional health department. In addition, they recommended having a consultation from the call center first before visiting a hospital if an individual had suspicious symptoms or had come in contact with a patient with COVID-19. This could prevent the spread of the virus from patients who are infected to health care providers.

Of the videos uploaded by independent users, two were “patient experience” videos. One video is filmed by a patient lying in a hospital bed who has COVID-19. The patient, who was having difficulty breathing, was trying to talk to the viewers about not taking any risks regarding COVID-19. The other video includes a survivor of COVID-19 who shares his personal story and experience with viewers. Viewers shared their opinions through the comments under these videos.

Thank you for sharing your story. I hope you will get better soon.

It looks like so much pain. It is so sad and scary.

When you go outside, please wear a mask. It is for our family and friends.

Science is not supposed to be a popularity contest, but governments should exert more effort to disseminate accurate and complete information via social media to ameliorate the negative health consequences of misinformation. Peer-reviewed or expert-approved videos are expected to provide credible medical information [61]. However, only a few of them that were uploaded by government agencies, universities, hospitals, and medical associations were included in this study because they did not rank within the 200 most viewed videos. The efforts of health care professionals cannot efficiently compete with malicious people and bots, who are able to automatically post millions of messages [62,63].

### Limitations

This study includes several limitations. First, this is a cross-sectional study, so it is limited to capturing YouTube scenes at a certain point in time. Common search terms or the most viewed videos may change over time, and longitudinal changes in video metrics such as views, likes, dislikes, or comments were not captured. Second, there is no validated tool for evaluating video-based medical information. Therefore, we adopted the evaluation tools commonly used in previous studies on online consumer health information. A comprehensive evaluation tool for the medical content of videos needs to be developed. Third, we could not tell whether watching a video clip led to a change in health behavior. For example, some people enjoy watching conspiracy videos to pass time and may be able to distinguish which stories are valid. Thus, they may end up following the guidelines of authorities regardless of their viewing such videos. More research is needed to evaluate the relationship between exposure to misinformation and health behaviors. Fourth, we did not collect any data on the viewers. All the YouTube video metrics were collected anonymously, so we could not grasp the viewers’ demographic characteristics.

### Conclusions

Misleading videos had more likes, fewer comments, and longer running times than useful videos. Korean-language YouTube videos on COVID-19 uploaded by different sources varied significantly in terms of reliability, overall quality, and title–content consistency, but the content coverage was not significantly different. Government-generated videos had higher reliability, overall quality, and title–content consistency than independent user–generated videos.

Although there is concern about the spread of misinformation via YouTube, the educational value of this website cannot be ignored. YouTube can be a powerful tool to keep the public informed during a crisis in a controlled and reassuring manner. However, to do so, accurate information must be made available on such platforms. Therefore, governments should have a stronger presence on social media and produce more online videos to reach a wider audience. First, to accomplish this, policy makers should support health institutions financially so they can use social networking platforms to their full potential. For example, an educational program could be developed to teach health care providers how to make YouTube videos and engage with the audience on social media. Second, health care professionals should cooperate with social media influencers, not compete with them, to reach more people. For example, the top-10 most subscribed YouTubers could create and upload a video in collaboration with a physician guest that provides a combination of entertainment and information on COVID-19. Thus, YouTube users would be able to obtain more high-quality information than false data. Third, compelling content should be used, as it can grab viewers’ attention. For example, visually attractive recordings can receive more views, and emotionally persuasive videos with exemplar stories are more likely to catch viewers’ attention than those with statistical evidence [64]. In this regard, effective communication via YouTube would contribute to reducing the risk of undesirable behavior, such as panic buying, and help the public distinguish between valid information and fake news. YouTube could serve as a rapid and inexpensive platform for reaching more people with accurate information during a public health crisis.

## Appendix

Multimedia Appendix 1 Modified DISCERN index.

Multimedia Appendix 2 The Journal of the American Medical Association Score benchmark criteria.

Multimedia Appendix 3 Global quality score.

Multimedia Appendix 4 Title–content consistency index.

Multimedia Appendix 5 Medical information and content index.

## Abbreviations

COVID-19: coronavirus disease
GQS: global quality score
ICC: intraclass correlation coefficient
JAMAS: Journal of the American Medical Association Score
KCDC: Korea Centers for Disease Control and Prevention
MICI: medical information and content index
TCCI: title–content consistency index
VPI: video power index
