# Supplementary material for: Evaluation of Korean-Language COVID-19–Related Medical Information on YouTube: Cross-Sectional Infodemiology Study
Source: J Med Internet Res. 2020 Aug 12;22(8):e20775. doi: 10.2196/20775 (PMC7425748; doi:10.2196/20775)
Supplement: Multimedia Appendix 1 [file jmir_v22i8e20775_app1.doc]

| Number of statements | Statements |
| --- | --- |
| a | Are the aims clear and achieved? |
| b | Are reliable sources of information used (i.e., publication cited and speaker is a certified physician)? |
| c | Is the information presented balanced and unbiased? |
| d | Are additional sources of information listed for patient reference? |
| e | Are the areas of uncertainty mentioned? |

Modified DISCERN index.
