# Supplementary material for: Evaluation of Korean-Language COVID-19–Related Medical Information on YouTube: Cross-Sectional Infodemiology Study
Source: J Med Internet Res. 2020 Aug 12;22(8):e20775. doi: 10.2196/20775 (PMC7425748; doi:10.2196/20775)
Supplement: Multimedia Appendix 2 [file jmir_v22i8e20775_app2.doc]

| Number of statements | Category | Statements |
| --- | --- | --- |
| a | Authorship | Authors and contributors, their affiliations, and relevant credentials should be provided. |
| b | Attribution | References and sources for all content should be listed clearly, and all relevant copyright information should be noted. |
| c | Disclosure | Website “ownership” should be prominently and fully disclosed, as should any sponsorship, advertising, underwriting, commercial funding arrangements and support, or potential conflicts of interest. |
| d | Currency | Dates when content was posted and updated should be indicated. |

The Journal of the American Medical Association Score benchmark criteria.
