# Supplementary material for: Evaluation of Korean-Language COVID-19–Related Medical Information on YouTube: Cross-Sectional Infodemiology Study
Source: J Med Internet Res. 2020 Aug 12;22(8):e20775. doi: 10.2196/20775 (PMC7425748; doi:10.2196/20775)
Supplement: Multimedia Appendix 3 [file jmir_v22i8e20775_app3.doc]

| Number of statements | Statements |
| --- | --- |
| 1 | Poor quality, poor flow of the site, most information missing, and not at all useful for patients |
| 2 | Generally poor quality and poor flow, some information listed, but many important topics missing, and of very limited use to patients |
| 3 | Moderate quality, suboptimal flow, some important information is adequately discussed but other is poorly discussed, and somewhat useful for patients |
| 4 | Good quality and generally good flow, most of the relevant information is listed but some topics not covered, and useful for patients |
| 5 | Excellent quality, excellent flow, and very useful for patients |

Global quality score.
