# Supplementary material for: Evaluation of Korean-Language COVID-19–Related Medical Information on YouTube: Cross-Sectional Infodemiology Study
Source: J Med Internet Res. 2020 Aug 12;22(8):e20775. doi: 10.2196/20775 (PMC7425748; doi:10.2196/20775)
Supplement: Multimedia Appendix 4 [file jmir_v22i8e20775_app4.doc]

Title–content consistency index.

| Number of statements | Statements |
| --- | --- |
| 1 | Eye‐catching thumbnail pictures and sensationalist headlines to capture attention, but video title does not match the content |
| 2 | Visually attractive thumbnail, strong emotionally appealing headlines, and only a little relevant information listed |
| 3 | Some relevant information listed, but large gaps between title and its content |
| 4 | Most of the relevant information discussed, but small gaps between title and its content |
| 5 | An excellent title for the content |
