# Supplementary material for: Evaluation of Korean-Language COVID-19–Related Medical Information on YouTube: Cross-Sectional Infodemiology Study
Source: J Med Internet Res. 2020 Aug 12;22(8):e20775. doi: 10.2196/20775 (PMC7425748; doi:10.2196/20775)
Supplement: Multimedia Appendix 5 [file jmir_v22i8e20775_app5.doc]

| Number of statements | Statements |
| --- | --- |
| Each item is given 1 point, if mentioned in the video, with the maximum score of 5. | |
| **Prevalence** | |
| a | Number of confirmed or suspected cases reported in South Korea |
| b | Number of deaths reported in South Korea |
| c | Number of patients who are severely ill in South Korea |
| d | Number of survivors or patients who tested positive again after being declared recovered in Korea |
| e | Number of countries involved and current status in other countries |
| **Transmission and precautionary measures** | |
| a | Transmission (including droplet transmission, close contact transmission, ventilation, and regular disinfection) |
| b | Hygiene (including washing hands and using a mask) |
| c | Social distancing |
| d | Reinforcement of social measures to limit mass-gathering events (including postponement of school opening, closure of religious and sports establishments, and cancellation of shows and festivals) |
| e | Self-isolation |
| **Signs and symptoms** | |
| a | Fever |
| b | Respiratory symptoms (including cough, sputum, sore throat, runny nose, and shortness of breath) |
| c | Myalgia, fatigue, lethargy, and chest discomfort |
| d | Abdominal discomfort and diarrhea |
| e | Loss of smell or taste |
| **Testing** | |
| a | Mentions test is possible |
| b | Mentions the 1,339 call center to get detailed information |
| c | Mentions screening centers (including drive-through screening, community health center, and designated public relief hospitals) |
| d | Mentions the guidelines for testing |
| e | Explains how this test is done (including how much the test costs and when the test result will come out) |
| **Treatment and outcome** | |
| a | Triage people who test positive according to their severity (self-isolation, living care centers, and hospitals) |
| b | Factors influencing severity (smoking, hypertension, diabetes mellitus, malignancy, cardiovascular disease, lung disease, and age) |
| c | Treatment is supportive, but some medications may be used, such as antimalarial and anti-human immunodeficiency virus agents. |
| d | Vaccination not currently available |
| e | Reactivation in cured patients |

Medical information and content index.
